# Challenges in the Development of an Immunochromatographic Interferon-Gamma Test for Diagnosis of Pleural Tuberculosis

**DOI:** 10.1371/journal.pone.0085447

**Published:** 2013-12-23

**Authors:** Claudia M. Denkinger, Yatiraj Kalantri, Samuel G. Schumacher, Joy S. Michael, Deepa Shankar, Arvind Saxena, Natarajan Sriram, Thangakunam Balamugesh, Robert Luo, Nira R. Pollock, Madhukar Pai, Devasahayam J. Christopher

**Affiliations:** 1 Division of Infectious Diseases, Beth Israel Deaconess Medical Center, Boston, Massachusetts, United States of America; 2 McGill International TB Centre & Department of Epidemiology, Biostatistics, and Occupational Health, McGill University, Montreal, Quebec, Canada; 3 Tulip Diagnostics, Goa, India; 4 Department of Microbiology, Christian Medical College, Vellore, India; 5 Department of Pulmonary Medicine, Christian Medical College, Vellore, India; 6 Department of Pathology, Stanford University, Stanford, California, United States of America; 7 Department of Laboratory Medicine, Boston Children’s Hospital, Boston, Massachusetts, United States of America; National Institute for Infectious Diseases (L. Spallanzani), Italy

## Abstract

Existing diagnostic tests for pleural tuberculosis (TB) have inadequate accuracy and/or turnaround time. Interferon-gamma (IFNg) has been identified in many studies as a biomarker for pleural TB. Our objective was to develop a lateral flow, immunochromatographic test (ICT) based on this biomarker and to evaluate the test in a clinical cohort. Because IFNg is commonly present in non-TB pleural effusions in low amounts, a diagnostic IFNg-threshold was first defined with an enzyme-linked immunosorbent assay (ELISA) for IFNg in samples from 38 patients with a confirmed clinical diagnosis (cut-off of 300pg/ml; 94% sensitivity and 93% specificity). The ICT was then designed; however, its achievable limit of detection (5000pg/ml) was over 10-fold higher than that of the ELISA. After several iterations in development, the prototype ICT assay for IFNg had a sensitivity of 69% (95% confidence interval (CI): 50-83) and a specificity of 94% (95% CI: 81-99%) compared to ELISA on frozen samples. Evaluation of the prototype in a prospective clinical cohort (72 patients) on fresh pleural fluid samples, in comparison to a composite reference standard (including histopathological and microbiologic test results), showed that the prototype had 65% sensitivity (95% CI: 44-83) and 89% specificity (95% CI: 74-97). Discordant results were observed in 15% of samples if testing was repeated after one freezing and thawing step. Inter-rater variability was limited (3%; 1out of 32). In conclusion, despite an iterative development and optimization process, the performance of the IFNg ICT remained lower than what could be expected from the published literature on IFNg as a biomarker in pleural fluid. Further improvements in the limit of detection of an ICT for IFNg, and possibly combination of IFNg with other biomarkers such as adenosine deaminase, are necessary for such a test to be of value in the evaluation of pleural tuberculosis.

## Introduction

Extrapulmonary TB (EPTB) accounts for approximately 25% of all TB, and poses major diagnostic challenges. Pleural TB is the second most common manifestation of EPTB after lymph node TB [1,2]. Existing diagnostic tests have inadequate accuracy and turnaround time, and require special expertise for sample acquisition and interpretation of results. A pleural fluid aspiration rarely yields a definite diagnosis. A biopsy of the pleural tissue for histology and culture is considered the diagnostic gold standard, but may still be falsely negative in 10% to 20% of cases [3,4]. Nucleic acid amplification tests (NAATs) for evaluation of TB in pleural effusions appear to have high specificity (98%) but relatively low sensitivity (62%) [5]. Xpert MTB/RIF (Cepheid Inc., Sunnyvale, CA), a recently developed NAAT, similarly had low sensitivity (25-50%) across a number of studies on pleural fluid (and one study on pleural biopsy), with consistently high specificity [6-8]. 

Biomarkers present in pleural fluid have been evaluated for the diagnosis of pleural TB. More than 100 studies, summarized in meta-analyses, indicate that adenosine deaminase (ADA) and interferon-gamma (IFNg) are accurate biomarkers of pleural TB [9,10]. ADA is released by activated lymphocytes and macrophages and is a non-specific marker of inflammation. Meta-analyses show that ADA (measured by its enzymatic activity, at varying cut-points) in pleural fluid specimens is 88–100% sensitive and 83–97% specific for the diagnosis of pleural TB [3,10-15]. Measurement with immunologic methods (e.g. enzyme-linked immunosorbent assay (ELISA) [16]) has not been explored for the detection of ADA in pleural fluid. 

IFNg is a soluble cytokine that is secreted by Th1 cells, cytotoxic T cells and NK cells and has pro-inflammatory properties [17]. Meta-analyses show that free, unstimulated IFNg in pleural fluid, as measured by ELISA or radioimmunoassay with levels above a pre-determined cut-off (varying from 1.5-300pg/ml) in the different studies is 89–96% sensitive and 96–97% specific for the diagnosis of pleural TB [3,9,12]. 

While these meta-analyses showed substantial heterogeneity and publication bias was possible, the observed accuracy of these biomarkers supports their use in the diagnosis of pleural TB. However, available tests for their measurement currently can only be performed in a laboratory. Therefore, we explored the feasibility of developing, in India, a low-cost (less than US$1), multiplexed point-of-care (POC) lateral-flow immunochromatographic test (ICT) for pleural TB based on IFNg and ADA. ICTs are extensively used for diagnosis of various infectious diseases (e.g. syphilis, malaria) and have revolutionized the care of HIV by facilitating rapid diagnosis at the POC [18,19]. For TB no such test exists, but a test could conceivably improve the care of patients with pleural TB. Development of such a test in a high-burden country would also build on and expand existing capacity, allow production at high volume and low cost (because of lower production cost) and shorten delivery pathways.

## Methods

This project was a collaboration between Tulip Diagnostics (Goa, India), Christian Medical College (“CMC”; Vellore, India) and the McGill International TB Center (McGill University, Montreal, Canada). The institutional review boards (IRB) of CMC and McGill University Health Centre (Montreal, Canada) approved the study. Patients who met the inclusion criteria (suspected to have TB pleural effusion, at least 18 years of age, able and willing to give informed consent) were explained the scope of the study, and written informed consent was obtained (IRB-approved consent form available in English, Hindi and Bengal).

### Derivation and validation cohort

**Figure 1:**
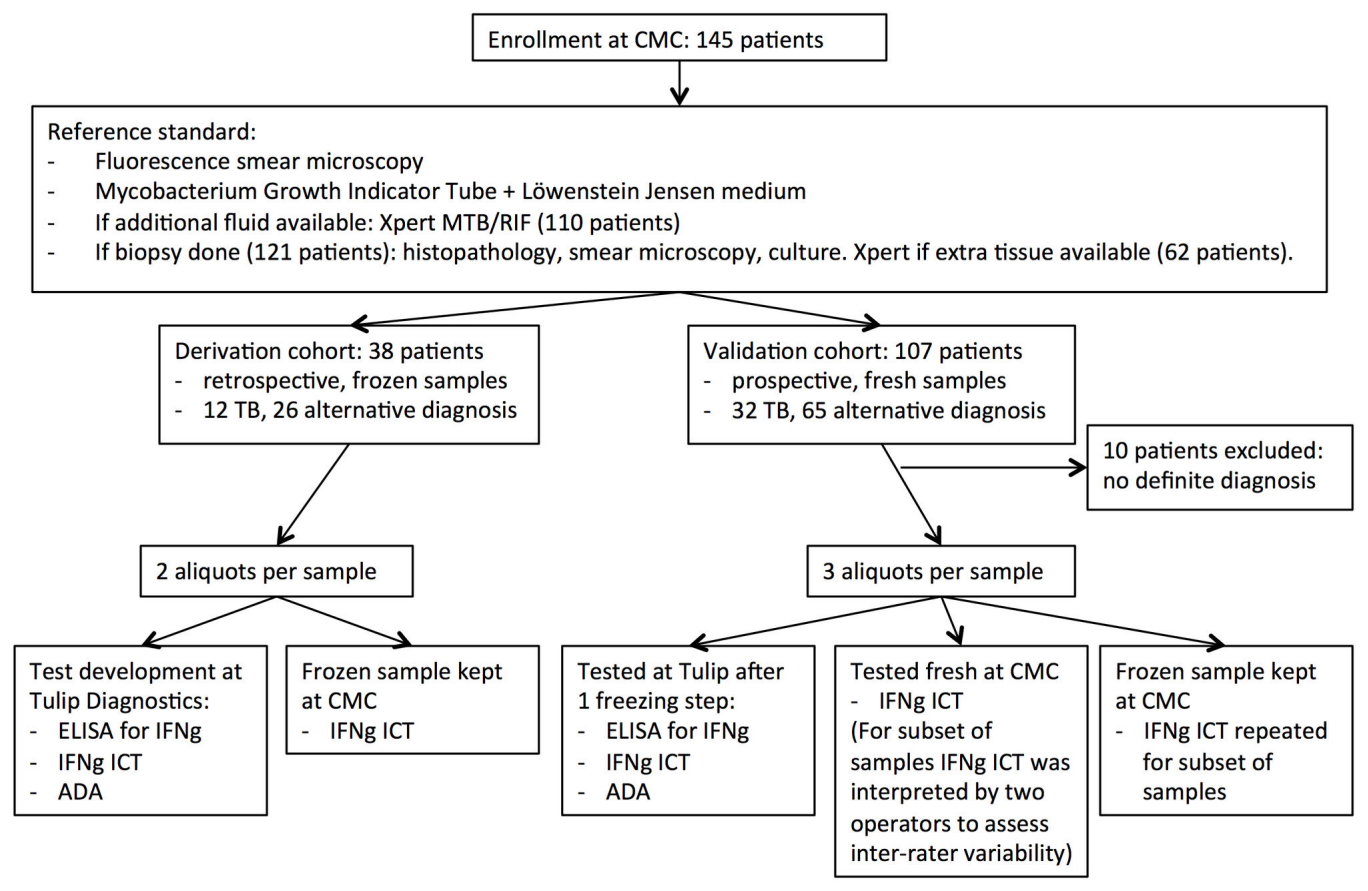
Study flow and location of testing. Legend: CMC=Christian Medical College; TB=tuberculosis; ELISA=enzyme-linked immunosorbent assay; ICT= lateral flow, immunochromatographic test; IFNg=interferon gamma; ADA=adenosine deaminase.

All patients enrolled in the study underwent thoracentesis (as part of their routine clinical care) in the pulmonary outpatient clinic or the pulmonary medical ward at CMC for evaluation of suspected pleural TB. When clinically indicated and safely feasible, a pleural biopsy was also performed. Figure 1 outlines the study flow and the location of testing performed at CMC versus Tulip. Information on demographics, co-morbidities, presenting symptoms and results of routine diagnostic evaluation was obtained from the chart. 

For test development, discarded, frozen pleural fluid specimens (collected within three months prior to the study) from patients who had either a confirmed diagnosis of pleural TB or a confirmed alternative diagnosis were used (derivation cohort). The frozen sample was thawed and divided into two aliquots. One was sent frozen to Tulip Diagnostics for test development and the remaining sample was kept at CMC (frozen at -80 degrees Celsius; Figure 1).

For the validation cohort, we prospectively enrolled 107 consecutive adult patients between August 2012 and May 2013 at CMC. Characteristics of the validation cohort are provided in Table 1. Pleural TB was suspected based on clinical symptoms and radiographic evidence of a pleural effusion. Clinicians used a Likert scale to rate their clinical suspicion for TB (prior to test results becoming available) as “not likely,” “likely” or “very likely.” The collected samples were aliquoted into three specimens. One aliquot was tested fresh at CMC; the second was frozen (at -80 degrees Celsius) and stored at CMC; and the third was sent frozen to Tulip (Figure 1). 

**Table 1:** Characteristics of patients in validation cohort.

Variables		n^*^	%
Subjects total		107	100
Age categorized			
	15-29	19	18
	30-49	40	37
	>50	47	44
Gender			
	Female	20	19	
	Male	86	81
	Fever	47	44
	Cough	76	71
	Hemoptysis	9	8
	Chest pain	61	57
	Shortness of breath	83	78
	Weight loss	45	42
	Night sweats	10	9
	Unilateral effusion	102	95
	Bilateral effusion	5	5
	Pulmonary infiltrates suggestive of tuberculosis	39	36
	Cavitations	1	1
	Human immunodeficiency virus (HIV) infection	0	0
	Diabetes	28	26
	Malnutrition	2	2
	End-stage renal disease	4	4
	History of malignancy	10	9
	Treatment with immunosuppressive medications	7	7
	Congestive heart failure	2	2
	Rheumatologic disease	2	2
History of active tuberculosis		22	21
Close contact with tuberculosis patient		4	4
Clinical diagnosis at first evaluation (without test results)			
	Alternative diagnosis more likely than tuberculosis	38	35
	Tuberculosis likely	35	33
	Tuberculosis very likely	34	32

Presenting symptoms

Radiographic findings

Co-morbidities

^*^Total number of subjects in validation cohort

One operator (YK) performed the IFNg ELISA/ICTs and ADA testing at Tulip. Two trained operators did the ICT testing at CMC at the POC in the clinic. For a subset of samples, both operators at CMC interpreted the ICT independently to assess inter-rater variability (Figure 1). The operators at the two testing sites (CMC and Tulip) were blinded to the reference standard and to the results of the tests at the other site.

### Reference standard

All pleural fluid specimens were processed with routine diagnostics in the CMC microbiology laboratory. Testing included fluorescence smear microscopy, liquid culture (Mycobacterium Growth Indicator Tube, MGIT, Becton Dickinson, Sparks, MD, USA) and solid culture (Löwenstein Jensen medium) for both the derivation and validation cohort. If additional fluid was available, an Xpert® MTB/RIF assay was also done at CMC (”Xpert;” Cepheid, Sunnyvale, USA) [7]. Pleural tissue, if obtained, was evaluated with histopathology, smear microscopy, and culture. Xpert was also done on tissue biopsy if sufficient sample was available [6]. Results of the different components of the reference standard for the patients in the derivation and validation cohorts are provided in Table 2.

**Table 2:** Results for different tests (within reference standard) performed on pleural fluid and pleural tissue.

**Diagnostic test results contributing to the diagnosis**		**n**	**Derivation cohort**		**Validation cohort**	
**Tuberculosis**		44	12		32*	
	Positive pleural tissue culture	9	1		8	
	Positive pleural fluid culture	1	0		1	
	MTB identified at other site	2	0		2	
	Histopathology with granulomas	38	11		27	
	Positive Xpert in pleural fluid	4	0		4	
	Positive Xpert in pleural tissue	2	1		1	
**Alternative diagnosis**		91	26		65*	
	Definite malignancy by pathology or cytology	47	11		36	
	Negative pleural tissue culture and histopathology	31	11		20	
	Other confirmed diagnosis (e.g. empyema)	13	4		9	

^*^ 10 patients without definite clinical diagnosis

We considered the diagnosis of pleural TB confirmed if smear, culture or Xpert on pleural tissue or fluid was positive for *Mycobacterium tuberculosis* (MTB), histopathology of pleural tissue identified granulomas, or MTB was present in any other respiratory sample (e.g. sputum, endobronchial or transbronchial biopsy). Pleural TB was ruled out if either histopathology was diagnostic for malignancy or both pleural tissue culture and histopathology were negative for TB. A definite alternative diagnosis (e.g. empyema or malignancy) was necessary for a sample to be included in the derivation cohort. A chart review was done for patients in the validation cohort with presumed false-positive results on the index test (but without a definite alternative diagnosis) but no additional TB cases were identified in follow-up. 

### Lateral-flow immunochromatographic test: principle of test, procedure and interpretation

The ICT utilizes the principle of immunochromatography and is a two-site immunoassay performed on a membrane. As the test sample flows through the membrane assembly of the test device the colored monoclonal anti-interferon gamma (IFNg) colloidal gold-conjugate complexes with the IFNg in the sample. The sample then moves further on the membrane to the test region, where it is immobilized by a monoclonal anti-IFNg antibody coated on the membrane leading to the formation of a pink/purple colored band, which confirms a positive test result. Absence of this colored band in the test region indicates a negative test result. The unreacted conjugated antibodies move further on the membrane and are subsequently immobilized by the goat anti-mouse antibody coated on the membrane at the control region, forming a pink/purple band. This control band serves to validate the test results.

**Figure 2:**
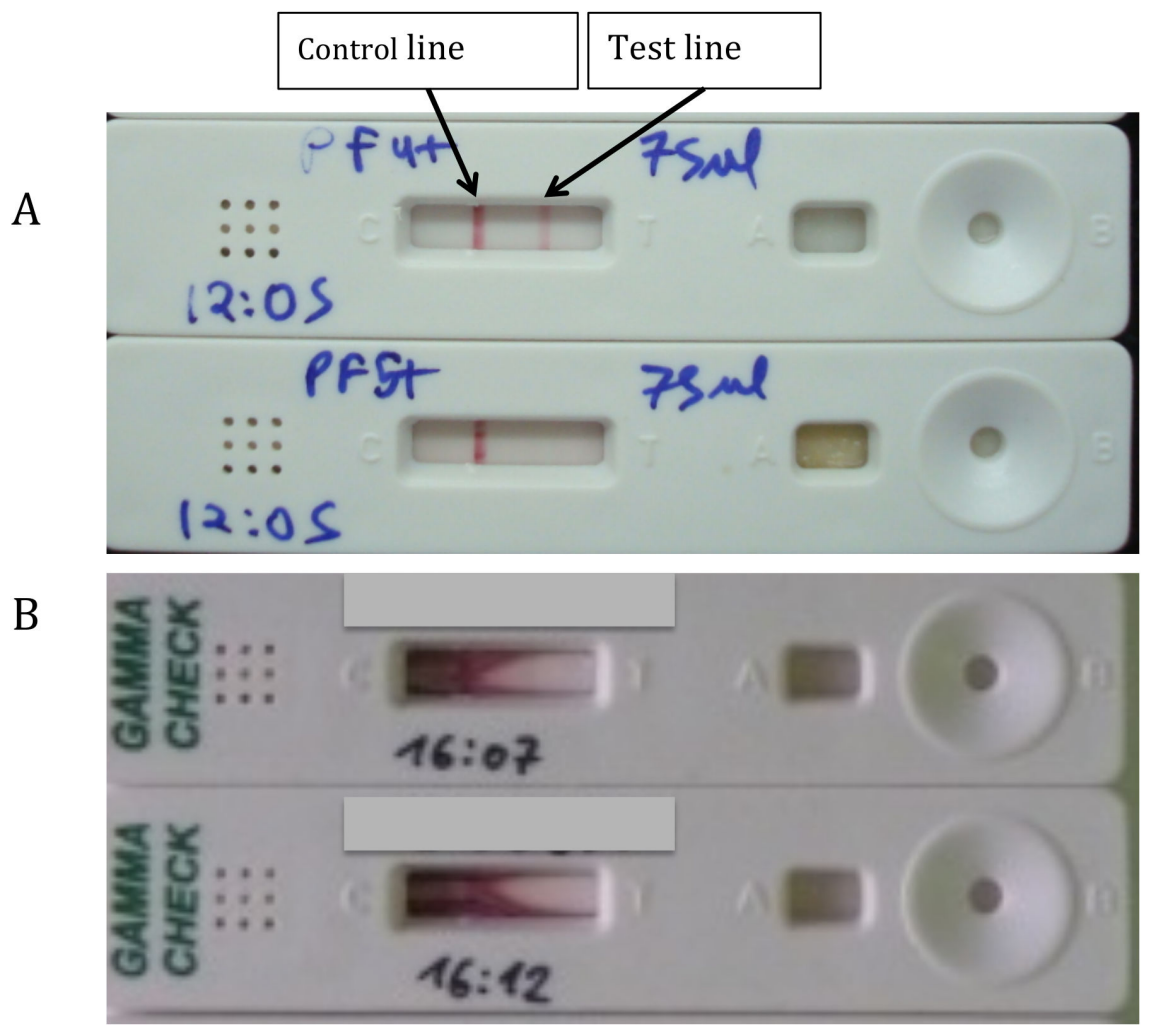
IFNg lateral flow test. (A) Initial results at Tulip with clearly defined lines indicating a positive (top) and negative (bottom) test. (B) Initial results at clinical evaluation site (CMC) with smearing of sample and incomplete advancement.

**Figure 3:**
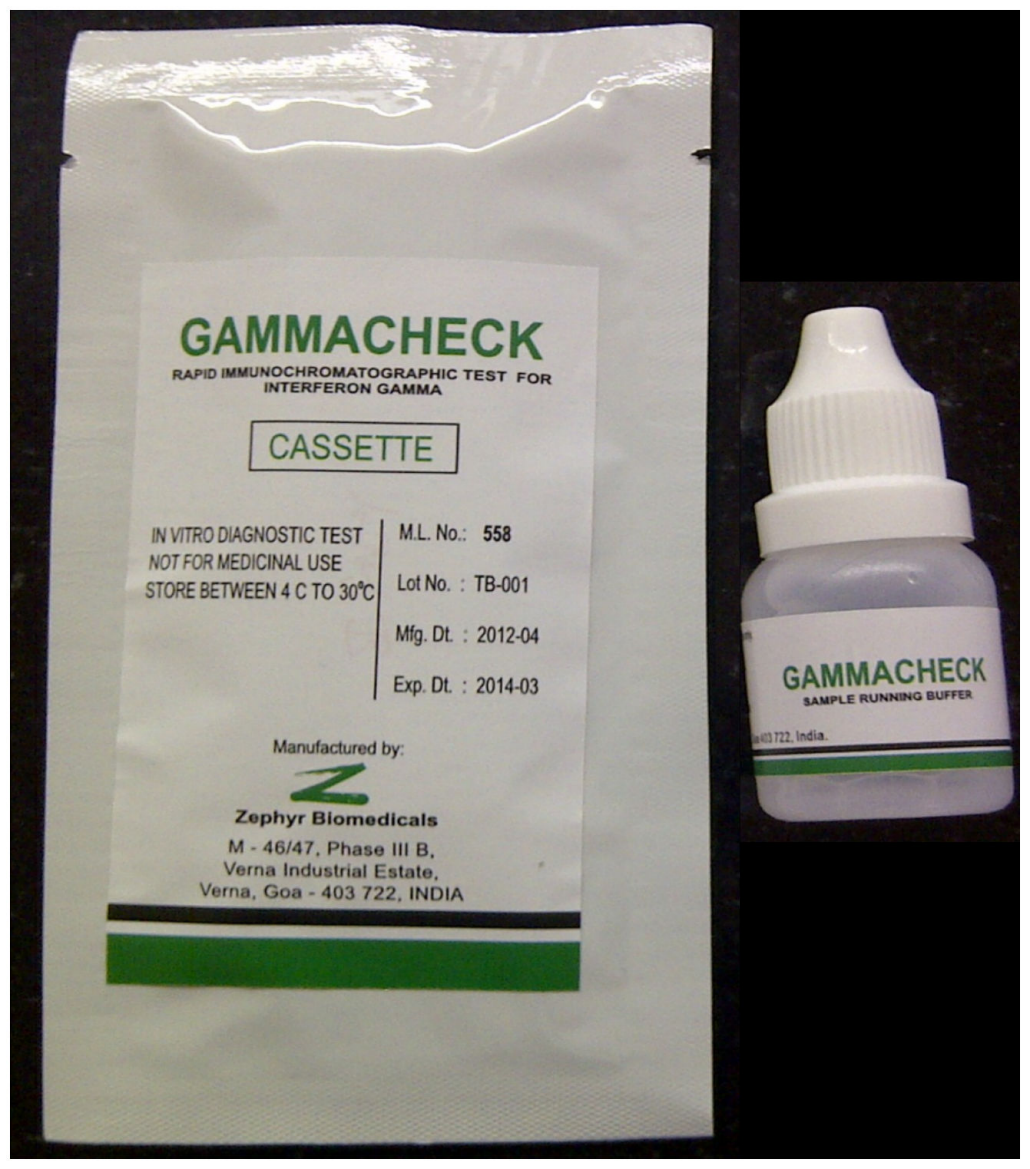
Prototype of the IFNg lateral flow test (named Gammacheck).

The procedure includes four steps: 1) adding specimen (50 µl) to the well marked “A” (see Figure 2); 2) adding sample running buffer to the well marked “B” (see Figure 2); 3) waiting 30 minutes; and 4) interpreting results.

The test is interpreted as “negative“ if only one pink/purple band appears at the control region “C” (see Figure 2). The test is interpreted as “positive” if in addition to the control band a pink/purple band (of any intensity) appears at the test region “T” (see Figure 2). The test is interpreted as “invalid” if the control band does not appear or if the band is incomplete (i.e. does not span the entire width of the testing field).

### Development of the ICT – 1^st^ prototype

The platform development was done at Tulip Diagnostics, one of the largest diagnostics manufacturers in India with extensive experience in the development of ICTs. Commercial antibodies against IFNg and ADA were obtained to first develop an ELISA and then a lateral flow test using standard principles of immunochromatography [19]. 

Of antibodies against ADA tested, no antibody was available that detected ADA in the quantities present in pleural fluid (around 30-60 IU/L measured by enzymatic activity) [3,10,12]. Thus, a decision was made early on to focus on an ICT with IFNg as the only biomarker. A list of ADA and IFNg antibodies tested, including suppliers and characteristics, is shown in Table S1.

The pair of commercially available monoclonal antibodies for ELISA from Mabtech (Nacka Strand, Sweden) provided the best and most consistent results for detection of IFNg by ELISA. Because IFNg is commonly present in non-TB pleural effusions in low amounts, a cut-off for positivity was established at Tulip Diagnostics based on the performance of the ELISA (with optical density measurements) on frozen samples from in patients with either confirmed TB (n=12) or an alternative diagnosis (n=26) in the derivation cohort from CMC (Figure 1). A cut-off of 300pg/ml as measured by ELISA resulted in the best sensitivity and specificity (94% and 93%, respectively). Values above 300pg/ml were deemed diagnostic for pleural TB (of note, this value is at the higher end of the range of cut-off values reported in the literature) [9]. 

Subsequently, the ICT prototype (called Gammacheck, Figure 3) was developed at Tulip Diagnostics using antibodies from Hytest (Turku, Finland) as these provided the best results on the ICT platform (Table S1). The antibody concentrations on the ICT were adjusted until values above 5000pg/ml would result in a positive test line. A lower limit of detection was not feasible on the ICT platform with this antibody pair. 

### Statistical analysis

We performed all the analyses with Stata, Version 12 (Stata Corp, Texas, USA). The analysis and reporting followed the Standards for the Reporting Diagnostic Accuracy (STARD) [20].

## Results

### Initial evaluation of 1^st^ prototype

Initial results under optimized conditions at Tulip Diagnostics, using aliquots of the same samples that were used to define the cut-off for the ELISA (derivation cohort), showed a sensitivity of 69% (95% confidence interval (CI): 41-89) and specificity of 100% (95% CI: 82-100%) in comparison to the ELISA. Testing was then performed in the clinical setting (at the CMC laboratory) on frozen aliquots from the same samples (from the derivation cohort) as well as on newly collected fresh samples (from a subset of the validation cohort), following the standard operating procedure for the ICT provided by Tulip Diagnostics. While the sensitivity and specificity on fresh samples at CMC were lower than what was observed at Tulip diagnostics (sensitivity 50% [95% CI: 16-84%], specificity 90% [95% CI: 67-99%], the confidence interval included the values observed in the original test development. However, in contrast to the 5% invalid results observed on initial testing at Tulip Diagnostics, 30% of results were invalid on testing of samples at CMC (Figure 2B). These invalid results were hypothesized to be related to the substantially higher ambient temperature (>30°C) and humidity at CMC (compared to the air-conditioned rooms at Tulip Diagnostics). In addition, we found that blood contamination of pleural fluid was associated with impaired flow when samples were tested fresh (rather than frozen). 

### Optimization and evaluation of 2^nd^ prototype

Further optimization work was thus done at Tulip Diagnostics in order to develop a second prototype. An additional layer was integrated onto the nitrocellulose membrane to reduce the evaporation of chase buffer at high temperatures and phytohemagglutin was added to the release pad to ensure adequate flow even in the presence of blood. 

The evaluation of the second prototype was done at CMC on 72 prospectively collected fresh samples from the validation cohort (in a non-air-conditioned room). An aliquot of each sample was frozen and shipped to Tulip Diagnostics for retesting with the prototype in addition to testing with the IFNg ELISA and with an enzymatic assay for ADA (ADA-MTB, Tulip Diagnostics) (Figure 1). 

The invalid rates of the second prototype were substantially improved: 3% at CMC (compared to 30% for the first prototype) and 0% at Tulip. In comparison with the ELISA as a reference standard, the performance of the ICT was as follows: sensitivity was 69% (95% CI: 51-83) at Tulip and 59% (95% CI: 41-75) at CMC and specificity was 94% (95% CI: 81-99) both at Tulip and at CMC. The test could not be performed on two samples at Tulip as the sample was too viscous after freezing and did not migrate along the membrane (Table 3).

**Table 3:** Comparing 2^nd^ prototype of lateral flow assay to ELISA results.

**Location**	**Specimen Condition**	**Sensitivity**	**Specificity**	**Invalid results**
**Tulip**	Frozen	24/35 (69%)	33/35 (94%)	0/70 (0%)**^*#*^**
**CMC**	Fresh	20/34 (59%)	34/36 (94%)	2/72 (3%)

^#^ 2 samples with insufficient amount to be retested

If the comparison was made to a clinical reference standard (definite diagnosis available for 64 patients out of 72), the ELISA had a sensitivity of 94% (95% CI: 79-99) and a specificity of 84% (95% CI: 73-92) in the validation cohort. The second prototype in comparison had a sensitivity of 65% (95% CI: 44-83) and a specificity of 89% (95% CI: 74-97) on fresh samples at CMC and a sensitivity of 76% (95% CI: 55-91) and specificity of 86% (95% CI: 71-95) after one freezing step at Tulip (Table 4).

**Table 4:** Comparing 2^nd^ prototype of lateral flow assay with clinical diagnosis^+*^.

**Location**	**Specimen Condition**	**Sensitivity**	**Specificity**	**Invalid results**
**Tulip**	Frozen	19/25 (76%)	31/36 (86%)	1/62 (2%)**^*#*^**
**CMC**	Fresh	17/26 (65%)	32/36 (89%)	2/64 (3%)

***^+^*** Diagnosis of TB confirmed if smear, culture or Xpert on pleural tissue or fluid was positive for *Mycobacterium tuberculosis* (MTB), histopathology of pleural tissue identified granulomas or MTB was present in any other sample (e.g. sputum, endobronchial biopsy); ^*^ 8 out of 72 patients without confirmed clinical diagnosis; ***^#^*** 2 samples tested at CMC with insufficient amount to be retested at Tulip

### Added value of ICT for clinician decision-making

Clinicians identified all TB patients that tested positive with the second prototype of the ICT as either “likely” or “very likely” to have TB based on clinical suspicion alone. Only for two patients ultimately identified as having TB did providers have a low suspicion of TB. These two patients also tested negative with the ICT.

The second prototype of the IFNg ICT identified five additional cases of TB confirmed by the clinical reference standard that would have not been identified by standard biochemical analysis (of the pleural fluid) only (i.e. lymphocytic predominance and an elevated ADA). However, it also was falsely positive in two cases where the standard biochemical analysis would have been true negative. For 38 out of 64 patients, the results of the biochemical analysis and the IFNg were concordant and correctly identified the diagnosis (27 true negative and 11 true positive). For 4 patients the results were concordant but incorrect (2 false negative and 2 false positive). Out of the remaining 22 patients, 15 had discordant results (IFNg ICT correct for 8 cases), for 2 the IFNg ICT failed and for 5 patients, the biochemical analysis with cell count and ADA was incomplete. 

### Discrepancy between results

Fifteen percent (10 samples) of results of the second prototype of the ICT as performed at CMC versus at Tulip were discrepant, with the majority being positive at Tulip and negative at CMC. This raised concerns about the freezing step (that occurred prior to testing at Tulip but not for testing of fresh samples at CMC) having an effect on the amount of IFNg in the pleural fluid. When testing with the 2^nd^ prototype was repeated at CMC on the frozen aliquots of 32 samples (note: no surplus frozen sample was available for 3 out of the 10 discordant samples), the discordance was resolved for three out of seven discordant samples included in this subset of samples and no new discordance emerged. 

Inter-rater variability in the interpretation of the results was also considered. However only 1 result out of 32 read by two independent readers at CMC was read differently between the two readers (i.e. interpreting the visibility of the lines differently, which resulted in a positive result read by one reader and a negative result by the other). This one discordant result between the CMC operators occurred in one of the seven samples for which results at CMC vs. Tulip were discordant, suggesting a possible borderline result. Three of the seven discordant (CMC vs. Tulip) results remained unexplained and are likely due to variability in the test performance. Alternatively, differences in test accuracy may have been due to differences in the temperature at which the test was performed in the two sites.

Given the limited performance of the test, and the limitations of further research and development possible to optimize the performance characteristics, a No-go decision was made and we decided to stop further investment into the project.

## Discussion

Improved diagnostics for pleural TB are urgently needed and biomarkers, such as IFNg and ADA, have been identified as having good sensitivity and specificity. However, testing via currently available procedures requires a laboratory and technical expertise. A test that rapidly yields a result and can be done at the POC, even with imperfect sensitivity, would be a step forward [3,4]. 

In this study we aimed to develop such a test for IFNg. The results show that an ICT for IFNg with a lower limit of detection of 5 ng/ml has limited sensitivity and specificity. The high limit of detection could explain shortcomings in sensitivity of the ICT test [3,9,12]. Further research to improve the limit of detection of IFNg on an ICT platform should be considered.

Prior studies that evaluated IFNg as a biomarker for pleural fluid also almost exclusively tested frozen samples. This could have resulted in cell-lysis and further release of IFNg from the intracellular compartment into the pleural fluid and thus increased levels of IFNg [17]. While we were able to increase the clinical sensitivity of the test with a freezing step, the numbers evaluated were too small and further research on comparing fresh and frozen samples is needed. However, this finding could point towards a more general problem for research on biomarkers that have a relatively large intracellular compartment that may be released through cell-lysis with a freeze/thaw step, as most exploratory studies are done on frozen samples [21]. In addition, the discrepant readings observed between different test sites could be related to the specimen itself. Pleural fluid can be a very heterogeneous sample and consistency can change after a freezing step, which potentially results in increased variability. 

Interrater variability was also considered, but not found to be a big concern (3%). Difficulties in reading ICT results have been described for HIV rapid tests, due to the subjectivity of test interpretation when the test yields a faint line [22,23]. 

The difference between findings in our study in comparison to the published literature on the performance of IFNg for the diagnosis of pleural TB could be reflective of both optimism bias and publication bias. Optimism bias for data reported in package inserts has been described for many TB tests [24], in particular those that did not undergo independent evaluation and approval by credible agencies such as the Federal Drug Administration (FDA) and World Health Organization (WHO). Most of the time this is observed when small numbers of patients are evaluated, controls are used that are not reflective of the population in regular clinical practice and high-risk groups are not included [24]. Similar reasons could also be implicated in the potentially optimistic results for IFNg in the published literature [9,11,12]. Furthermore, publication bias is likely contributing to the overoptimistic summary estimates in the meta-analyses [21,25]. 

Several studies on pleural TB diagnosis have shown that due to limitations of individual markers, a combination of markers might be more useful [26,27]. Development of an ICT for ADA was not feasible in our hands based on the antibodies available. However, alternative detection methods for ADA on a POC platform or alternative biomarkers could be considered in combination with IFNg. 

In summary, an ICT for IFNg did not achieve adequate sensitivity and specificity for the diagnosis of pleural TB. Furthermore, the test still would require a thoracentesis, which limits its use to hospitals and large health care centers. Further research should be pursued to optimize the limit of detection of IFNg on an ICT platform or ideally to identify biomarkers in more easily accessible body compartments (e.g. urine or blood) for the diagnosis of pleural TB and EPTB in general.

## Supporting Information

Table S1 **Antibodies tested for product development.**
(DOCX)
