# Supplementary material for: Challenges in the Development of an Immunochromatographic Interferon-Gamma Test for Diagnosis of Pleural Tuberculosis
Source: PLoS One. 2013 Dec 23;8(12):e85447. doi: 10.1371/journal.pone.0085447 (PMC3871622; doi:10.1371/journal.pone.0085447)
Supplement: Table S1 — Antibodies tested for product development. (DOCX) [file pone.0085447.s001.docx]

**Challenges in the development of an immunochromatographic interferon-gamma test for pleural tuberculosis**

**- Supplemental information**

**Table S1: Antibodies tested for product development**

A. Interferon-gamma antibodies

| Supplier (Location) | Clonality | Host | Isotype |
| --- | --- | --- | --- |
| Mabtech (Nacka Strand, Sweden) | Monoclonal | Mouse | IgG1 |
| Mabtech (Nacka Strand, Sweden) | Monoclonal | Mouse | IgG1 |
| Hytest (Turku, Finland) | Monoclonal | Mouse | IgG1 |
| Hytest (Turku, Finland) | Monoclonal | Mouse | IgG1 |

B. Adenosine Deaminase

| Supplier (Location) | Clonality | Host | Isotype |
| --- | --- | --- | --- |
| Abnova (Taipei City Taiwan) | Monoclonal | Mouse | not reported |
| Abnova (Taipei City Taiwan) | Polyclonal | Rabbit | not reported |
| Abcam (Cambridge, UK) | Polyclonal | Rabbit | IgG |
| Abcam (Cambridge, UK) | monoclonal | Mouse | IgG1 |
